# Plasma Extracellular Vesicles as Liquid Biopsy to Unravel the Molecular Mechanisms of Cardiac Reverse Remodeling Following Resynchronization Therapy?

**DOI:** 10.3390/jcm12020665

**Published:** 2023-01-13

**Authors:** Frans A. van Nieuwenhoven, Blanche Schroen, Lucio Barile, Lars van Middendorp, Frits W. Prinzen, Angelo Auricchio

**Affiliations:** 1 Department of Physiology, Cardiovascular Research Institute Maastricht, Maastricht University, 6200 MD Maastricht, The Netherlands; 2 Department of Cardiology, Cardiovascular Research Institute Maastricht, Maastricht University, 6200 MD Maastricht, The Netherlands; 3 Laboratory for Cardiovascular Theranostics, Istituto Cardiocentro Ticino, 6900 Lugano, Switzerland; 4 Department of Cardiothoracic Surgery, Maastricht University Medical Center, 6200 MD Maastricht, The Netherlands; 5 Department of Cardiology, Istituto Cardiocentro Ticino, Ente Ospedaliero Cantonale, 6900 Lugano, Switzerland

**Keywords:** microRNA, extracellular vesicles, heart failure, cardiac resynchronization therapy, dyssynchrony

## Abstract

Cardiac resynchronization therapy (CRT) has become a valuable addition to the treatment options for heart failure, in particular for patients with disturbances in electrical conduction that lead to regionally different contraction patterns (dyssynchrony). Dyssynchronous hearts show extensive molecular and cellular remodeling, which has primarily been investigated in experimental animals. Evidence showing that at least several miRNAs play a role in this remodeling is increasing. A comparison of results from measurements in plasma and myocardial tissue suggests that plasma levels of miRNAs may reflect the expression of these miRNAs in the heart. Because many miRNAs released in the plasma are included in extracellular vesicles (EVs), which protect them from degradation, measurement of myocardium-derived miRNAs in peripheral blood EVs may open new avenues to investigate and monitor (reverse) remodeling in dyssynchronous and resynchronized hearts of patients.

## 1. Introduction

Over the last quarter of a century, cardiac resynchronization therapy (CRT) has become a valuable addition to the treatment options for heart failure [1,2,3]. CRT is a unique therapy for treating patients with heart failure because it improves all aspects desired for those patients: it acutely increases cardiac pump function, increases exercise tolerance and, in the long run, leads to “reverse remodeling”, clinically defined as a reduction in left ventricular (LV) volumes. These changes are also associated with reduced hospitalization for heart failure and better survival. However, the benefit of CRT varies strongly between patients, ranging from an apparent adverse effect to complete normalization of ventricular pump function.

In addition, there is a remarkably poor correlation between the acute hemodynamic effect and the long-term reverse remodeling, suggesting that separate processes are involved in reverse remodeling in addition to hemodynamic loading. While reverse remodeling is clinically defined as a reduction in cavity volume, it is important to note that these volumetric changes are the consequence of molecular and structural changes in the myocardial tissue.

Abnormal, slow conduction of the electrical impulses in the ventricles, such as during both bundle branch blocks and ventricular pacing, create abnormal contraction patterns. These patterns are characterized by the mechanical unloading of early-activated regions and the overload of late-activated regions [4,5]. In dog models, left bundle branch block (LBBB) results in dyssynchronous activation and contraction followed by ventricular dilatation and asymmetric hypertrophy [6].

Data from transcriptome microarray analyses in dogs revealed profound regional heterogeneity in the expression of hundreds of genes, including microRNAs (miRNAs), in the dyssynchronous left ventricle [7,8]. Many of the differentially expressed genes are known to be involved in myocardial remodeling processes, including hypertrophy, fibrosis and cell-survival signaling. Application of CRT in these animal models of cardiac dyssynchrony caused the reversal of most of these myocardial remodeling processes and gene expression signatures [7,8,9,10,11].

Since measuring those changes requires invasive tissue sampling, very little is known about these molecular and structural processes from patients suffering from cardiac “dyssynchronopathy”. Recently, extracellular vesicles (EVs) have been considered a prominent and universal form of intercellular communication [12]. Fundamentally, all cells in the organism are thought to secrete EVs [13]. EVs transport bioactive molecules including miRNAs, non-coding RNAs that take part in intracellular signaling and regulation of cell function. miRNAs were found to be involved in myocardial remodeling processes, and circulating miRNAs appear to be linked to (reverse) remodeling of the myocardium in patients [14].

This article aims to review what is known about dyssynchrony-induced myocardial remodeling and its CRT-induced reversal, focusing on the question of whether EV-derived miRNAs from peripheral blood may be used as “liquid biopsy” for prediction and monitoring of CRT response in patients [15].

## 2. Myocardial Remodeling in Cardiac Dyssynchrony

Inappropriate cardiac electric signal conduction leads to maladaptive changes in electrophysiology, contractility and finally also clear structural abnormalities. Typically the ventricular activation time is prolonged, resulting in the broadening of the QRS complex [6]. Action potential duration (APD) is differentially affected in early- and late-activated regions of the heart, with evidence that locally different changes in APD are caused by the local differences in mechanical load (mechano-electrical feedback) [16,17]. Although some of these electrophysiological changes are a direct result of disturbed conduction, others are caused by changes in gene expression, or cellular localization, of ion channels and gap junction proteins [10,18]. CRT generally improves ventricular activation and shortens the duration of the QRS complex [19]. While acute QRS shortening is logical due to the improved electrical activation by biventricular pacing, QRS reduction in the longer run [20] may have additional explanations: (1) reduced cavity size by reverse remodeling; (2) reduced fibrosis; (3) normalization of expression of ion channels, including gap junction channels.

Structural changes observed in experimental LBBB models include ventricular dilatation and asymmetric hypertrophy, specifically in the region of late activation [19,21]. While isolated LBBB in otherwise healthy animals did not show extensive changes in the cardiac extracellular matrix (ECM), more severe experimental models of cardiac dyssynchrony (i.e., by adding rapid pacing) show extracellular matrix (ECM) accumulation and fibrosis [9,11,22]. These structural changes are accompanied by regionally different degrees of myocardial expression of paracrine factors such as osteopontin, transforming growth factor beta (TGFβ), connective tissue growth factor (CTGF) and B-type natriuretic peptide (BNP). In these experimental models of cardiac dyssynchrony, CRT partly reverses the structural and paracrine abnormalities [7,11,23]. Similarly, the majority of CRT patients (CRT responders) show a reduction in LV cavity size and hypertrophy [24,25]. In addition, some studies show reduced myocardial fibrosis following CRT in patients, as measured in tissue samples from the right ventricular side of the interventricular septum [26,27].

LV contractile function is decreased in experimental dyssynchrony [19] as well as in dyssynchrony patients [28,29]. The acute changes observed following a conduction disturbance are explained by immediate mechanical dyssynchrony leading to uncoordinated and less efficient myocardial contraction [30]. However, further deterioration of contractility during prolonged cardiac dyssynchrony is caused by alterations in cellular structure and function associated with altered expression of genes involved in calcium handling, adrenergic signaling and sarcomere structure, as reviewed recently [31,32]. The acute restoration of synchronous contraction by CRT also improves contractile function immediately, while further improvements over time are explained by reversed remodeling at the molecular and cellular levels [19,33,34,35].

## 3. miRNAs in Remodeling in Dyssynchronous Hearts

Recent evidence has shown that non-coding RNAs including miRNAs are important regulators of cardiac function [36,37,38]. miRNAs act mainly by regulating gene expression at the post-transcriptional level, either by directing their target mRNAs to be degraded or by inhibiting their translation. Several studies have shown that miRNAs are key players in heart remodeling, including hypertrophy and fibrosis, in in vivo disease models and in vitro [39,40,41,42]. Overexpression of miRNA-195 leads to cardiac pathological hypertrophy and heart failure in mice [43]. Cardiac-specific miRNAs collectively known as myomiRNAs (miRNA-208a, miRNA-208b, miRNA-499, miRNA-133, miRNA-1) affect myosin content and the function of the myocardium [42,44]. miRNA-208 is required for myocardial hypertrophy, fibrosis, and beta-MHC upregulation in cardiomyocytes under stress conditions [42]. miRNA-133 and miRNA-1 are downregulated in cardiac hypertrophy in mice and humans [45,46], while overexpression of both miRNAs inhibits cardiac hypertrophy [46,47,48]. In other transgenic models, inhibition of miRNA-133 causes significant cardiac hypertrophy and cardiac dysfunction [46]. Myocardial miRNA-29 [49] and miRNA-30 and -133 [44] have been shown to play a role in cardiac fibrosis.

In order to gain more insight into the role of miRNAs in dyssynchrony, in a dog model of LBBB, regional differences in the myocardial expression of miRNA-133 were investigated and found. Local downregulation in the left ventricular free wall (LVfw), which is mechanically overloaded in LBBB, coincided with hypertrophy and with increased expression of CTGF [7]. In addition, the introduction of CRT in this model lead to a normalization of the distribution of miRNA-133, suggesting a role for this miRNA in (reverse) remodeling in dyssynchrony and indicating that both hypertrophy and this miRNA are regulated locally. Interestingly, in this model, other well-known (myo)miRNAs such as miRNA-155, -199 and -499 were not affected, pointing to the specific involvement of miRNA-133 in this disease model, potentially pointing to different molecular mechanisms of the development of heart failure in the different causes of heart failure. Myocardial miRNA-29 and -30 were not affected either in the LBBB model [7].

**Figure 1:**
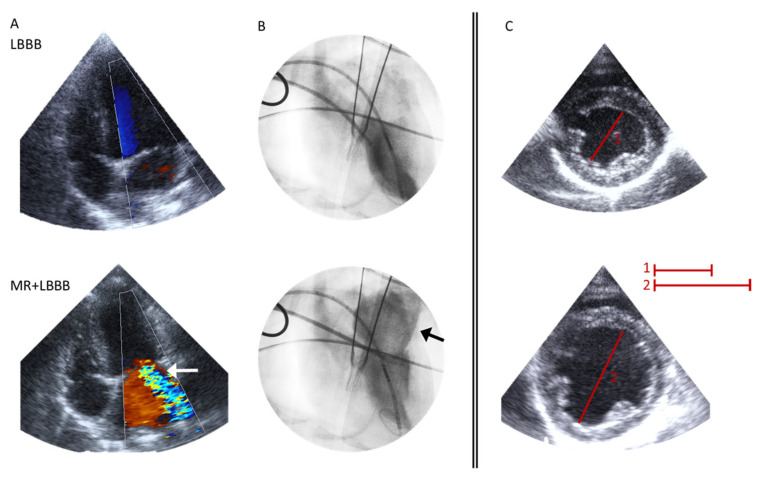
Evaluation of mitral regurgitation. (**A**,**B**) Representative perioperative recordings of a dog with LBBB (top panels) and MR + LBBB (bottom panels). (**A**) Four-chamber recording with color Doppler, showing a clear eccentric jet (white arrow) after ablation of chorda(e) in an MR + LBBB dog. (**B**) Fluoroscopic recording illustrating MR severity; black arrow indicates regurgitant flow into the left atrium. (**C**) Postoperative short-axis recording at week 16 (images have the same scale). Red lines show a clear increase in left ventricular internal diameter in diastole.

In order to investigate whether the difference in miRNA regulation between LBBB-induced cardiac remodeling and other animal models of cardiac remodeling may be explained by the lower degree of hemodynamic loading in LBBB animals, in a subsequent series of experiments, LBBB was combined with volume overload, as introduced by mitral regurgitation. Under fluoroscopic guidance, one or several chordae tendineae were grasped. The potential severity of MR was estimated by pulling on these chordae and evaluating the severity of the MR with the use of echocardiography [50]. As can be observed in Figure 1, the combination of MR and LBBB lead to severe LV dilatation.

Here we report yet unpublished data from studies comparing changes in LBBB and MR + LBBB animals. In the animals with MR + LBBB, the MR was created 4 weeks before LBBB was made. Both LBBB and MR + LBBB animals were subsequently followed for 16 weeks. While survival was 100% in LBBB animals, MR led to premature death in 5 out of 13 animals, and in the surviving animals, significant LV dilatation was observed (Figure 1), along with clinical symptoms of heart failure.

**Figure 2:**
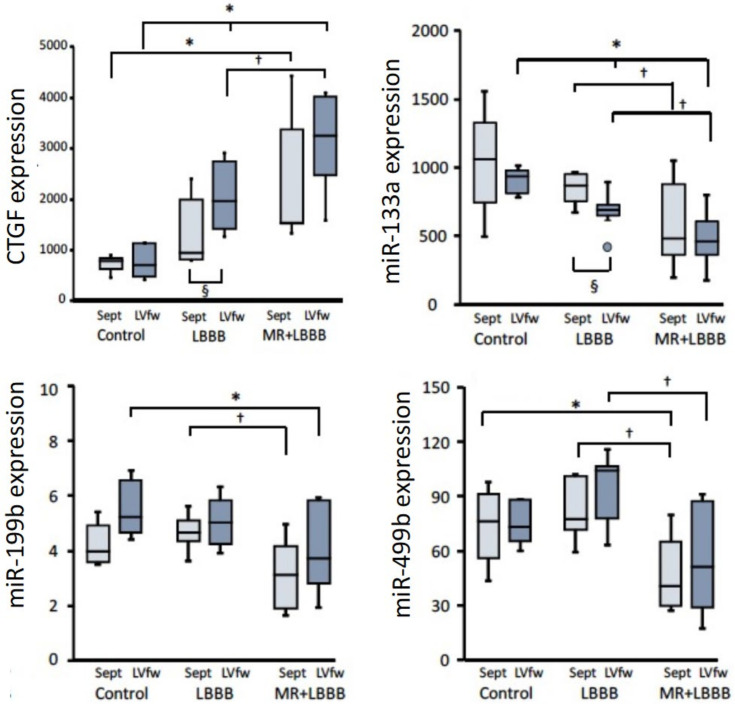
Boxplots of the expression of connective tissue growth factor (CTGF). and of microRNAs (miRs) related to hypertrophy. Dark blue boxes represent the LVfw and light blue boxes represent the septum. Data from 5 control, 8 LBBB and 8 MR + LBBB animals. * *p* < 0.05 vs. control; † *p* < 0.05 vs. LBBB; § *p* < 0.05 vs. septum using linear mixed-effect model analysis. Dashed lines indicate 100% (=median of control values in corresponding wall segment).

Figure 2 shows that in both LBBB and MR + LBBB animals, expression of the growth factor CTGF was significantly higher in the LVfw than in the septum and that the values were significantly higher in the MR + LBBB than in the corresponding tissues from the LBBB animals, indicating an additive effect of the loading conditions imposed by the LBBB and the MR. The hypertrophy-inhibiting miRNA-133 showed similar patterns, but now showed lower expression in the tissue submitted to the highest mechanical load. Less pronounced changes, if any, were observed for miRNA199 and -499. Still, expression of these two myo-miRNAs was significantly lower in MR + LBBB than in LBBB tissue, although differences within the same heart were not significant.

**Figure 3:**
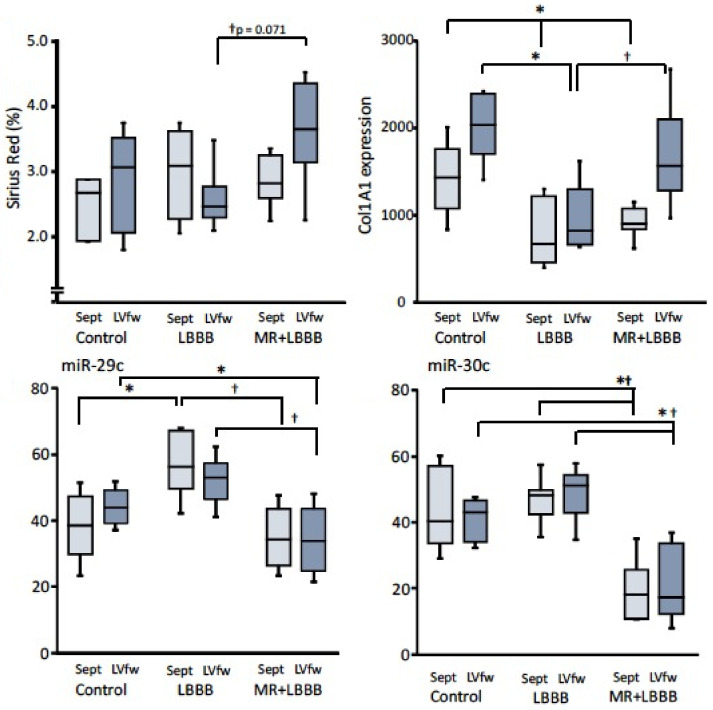
Upper panels: deposition of collagen (Sirius Red staining) and collagen-1A1 expression in control, LBBB and mitral regurgitation + LBBB (MR + LBBB) animals (same as in Figure 2). Lower panels: boxplots of the expression of miRs related to the extracellular matrix (miR-29c and miR-30c). Dark blue boxes represent the left ventricle free wall (LVfw) and light blue boxes represent the septum (Sept). * *p* < 0.05 vs. control; † *p* < 0.05 vs. LBBB. Dashed lines indicate 100% (=median of control values in corresponding wall segment).

Figure 3 shows that the anti-fibrotic miRNA-29 was upregulated in LBBB hearts, suggesting a corrective mechanism that tries to reduce fibrosis as a consequence of increased mechanical load. However, this miRNA was not upregulated in MR + LBBB hearts. In contrast, miR-30 was not abnormally expressed in LBBB but was significantly downregulated in MR-LBBB hearts. These downregulations in miRNA-29 and -30 were accompanied by increased expression of collagen and the *collage1A1* gene, especially in the LVfw (Figure 3). These data indicate that miR-133a is a very sensitive marker of (regional) loading in LBBB hearts whereas miR-199, -499 and -30 require more pronounced cardiac overload in order to become abnormally expressed. The data also demonstrate the additive effect of the two different kinds of mechanical load.

## 4. The Role of Extracellular Vesicles and Their Cargos in Mechanisms and Diagnosis of Cardiac Disease

The aforementioned data on miRNAs refer to myocardial expression and are hard to obtain in patients. However, at least some of the miRNAs from the heart are released by the heart in EVs [13,51]. Circulating miRNAs, including those released by cardiomyocytes, are mainly enclosed into EVs, which has the practical advantage, from a diagnostic perspective, that they are protected from enzymatic degradation [52]. Moreover, circulating cardiac-released miRNAs may have functional effects. Indeed, miR-21-3p is enriched in myofibroblast-derived EVs and causes repression of L-type calcium channel Cav1.2 levels in cultured rat cardiomyocytes [53]. Similarly, EV-enclosed miR-23a derived from atrial myocytes was found to impact M2 macrophage polarization and collagen production by atrial fibroblasts in a series of in vitro experiments [54]. It was suggested that this miRNA has great potential in the diagnosis and treatment of atrial fibrillation [55].

In relation to dyssynchrony and CRT, a few studies have shown evidence that circulating miRNAs may play an important role in the pathophysiology and possibly also in future diagnosis in this field.

Ben-Zvi et al. [56] measured levels of cardiac-specific miRNAs in peripheral and coronary sinus plasma of controls and CRT patients. CRT patients had higher levels of miRNA-125 and miRNA-133 in peripheral venous blood. Moreover, the CRT patients had higher levels of miRNA-125 and -133 in the coronary sinus than in peripheral blood, supporting the idea that the presence of these miRNAs is at least partly due to release from the heart. Interestingly, levels of miRNA-92 and miRNA-21 were lower in the coronary sinus, compared with the peripheral venous circulation, suggesting that some miRNAs are taken up by the heart, possibly contributing to regulation of myocardial function. In addition, McAloon et al. showed higher miRNA-133, -30 and -486 levels in the coronary sinus than in the peripheral blood of CRT patients.

Moscoso and colleagues [57] studied a small (*n* = 28) group of CRT patients, dividing them into responders and non-responders, based on the increase in LV ejection fraction. They found weak but significant inverse relations between baseline plasma miRNA-499 and CRT response. In addition, the CRT-induced reduction in miRNA-125 levels was related to CRT response, indicating that changes in circulating miRNA-125 levels reflect events within the myocardium.

The most extensive study was performed by Melman et al. [58]. These investigators started with assessing baseline levels of 766 plasma miRNAs in a discovery set of CRT patients (*n* = 12) with a variety of subsequent echocardiographic improvements at 6 months after CRT. Validation of candidate miRNAs was performed in 61 additional patients and confirmed that baseline plasma miRNA-30d was positively associated with a favorable echocardiographic CRT response. As further support of the cardiac origin, they showed that miRNA-30d was enriched in the coronary sinus blood of dogs with dyssynchronous heart failure. Furthermore, overexpression of miRNA-30d in cultured cardiomyocytes led to cardiomyocyte growth and protected against apoptosis by targeting the mitogen-associated kinase 4, a downstream effector of tumor necrosis factor. Further studies are required to reconcile the myocardial underexpression of miR-30c in the abovementioned study in MR + LBBB animals (Figure 3) with the miR-30d enrichment in the coronary sinus blood in the Melman study. Moreover, in our studies in the LBBB animals, not yet showing a change in miRNA-30c (yet), a significant benefit of CRT was still shown [6], raising the question of how specific miRNA-30 is for dyssynchronous heart failure.

Marfella et al. [59] performed a large study analyzing 84 heart failure-associated miRNAs in the serum of 81 patients with heart failure undergoing CRT, of which 55 were designated CRT responders after 12 months; 60 matched controls; and 15 healthy subjects. There were no statistical differences in miRNA levels at baseline between CRT responders and non-responders, and 24 miRNAs had lower circulating levels in patients with heart failure as compared to controls. Interestingly, 1 year after CRT, of the 24 miRNAs that were identified as having lower circulating levels in heart failure patients as compared to controls, 19 miRNAs seemed to show a restoration of their circulating levels. Among these, seven miRNAs previously implicated in cardiac hypertrophy, apoptosis and fibrosis showed a strong >5-fold increase in levels (miRNA-26b-5p, -145-5p, -92a-3p, -30e-5p, -423a-5p, -21-5p and -29a-3p), implying that these miRNAs might be involved in CRT-induced cardiac remodeling [59].

miRNA-30e-5p is a family member of the prime candidate of Melman et al. [58], miR-30d, sharing the same seed sequence. However, whereas Marfella and colleagues [59] report lower baseline circulating miR-30e-5p levels in patients with heart failure as compared to control patients, and an 8-fold increase in responders after 12 months of CRT, Melman et al. [58] show a decrease in miRNA-30d levels in responders as compared to non-responders at 6 months following CRT. Letters have been exchanged between these authors to further explore the meaning of these interesting findings [60,61]. Nevertheless, the consensus between both groups is that the miRNA-30 family is a central player in cardiac remodeling [40,62,63] upon CRT that requires further investigation in transgenic models to explore its exact role in the in vivo development of heart failure and reverse remodeling.

Interestingly, the changes in circulating miRNA profile in CRT responders in both the Melman and the Marfella studies were larger than those of the non-responders, both in the number of miRNAs and in the magnitude of changes. Non-responders in the Marfella study largely overlapped with the responders but involved only 5 of the 19 miRNAs that were restored in the responders, and those 5 showed lower restoration levels. In the non-responder group, only miRNA-885-5p was uniquely regulated; with lower levels at baseline in patients as compared to controls, and higher levels 1 year after CRT. This miRNA, implicated in fibrosis and apoptosis, might be further explored as a biomarker of CRT non-responsiveness.

Despite differences in approach and investigated miRNAs, the above studies indicate that levels of circulating vesicular miRNAs mirror changes in expression occurring in the myocardium. Therefore, serum- or plasma-derived EVs might realistically carry informative insights on organ function that is otherwise only accessible through invasive tissue biopsies. Moreover, as biomolecules stay more stable in EVs than freely in plasma, measuring biomarker content in EVs can obtain higher sensitivity than measuring biomarker content in biological fluids. It is plausible that purifying single subpopulations of endogenous cardiac-specific EVs, which to date remains a challenging issue, would further enhance the sensitivity and specificity of the biomarker. Another approach may be the use of artificial intelligence to identify patterns of miRNA presence in EVs that are indicative of heart failure and are predictive of CRT response and/or reflect molecular and structural changes in the heart with dyssynchronous heart failure and resynchronization.

## 5. Conclusions

Dyssynchronous hearts show extensive molecular and cellular remodeling. Evidence showing that at least several miRNAs play a role in this remodeling is increasing. A comparison of results from measurements in plasma and myocardial tissue suggests that plasma levels of miRNAs may reflect the expression of these miRNAs in the heart. Because many miRNAs released in the plasma are included in EVs, which protect them from degradation, measurement of myocardium-derived miRNAs in peripheral blood EVs may open new avenues to investigate and monitor (reverse) remodeling in dyssynchronous and resynchronized hearts. For example, EVs may improve the selection of patients for CRT by assessing a molecular substrate amenable to CRT, on top of an electromechanical substrate [64,65,66]. In addition, serial measurements in EVs may be used to monitor the molecular effects of CRT and potentially be used to adjust the therapy, such as AV delay and pacing site [67,68,69]. Finally, EVs may be helpful in assessing differences in CRT benefits between conventional biventricular pacing and the novel left bundle branch pacing [67,70].

## Data Availability

Experimental data are available upon request to the corresponding author.
